# Lunasin functionally enhances LDL uptake via inhibiting PCSK9 and enhancing LDLR expression *in vitro* and *in vivo*

**DOI:** 10.18632/oncotarget.20590

**Published:** 2017-08-24

**Authors:** Lili Gu, Yue Wang, Yaqiong Xu, Qinghua Tian, Gaoxin Lei, Cheng Zhao, Zhan Gao, Qin Pan, Wenfeng Zhao, Liu Nong, Shuhua Tan

**Affiliations:** ^1^ State Key Laboratory of Natural Medicines, School of Life Science and Technology, China Pharmaceutical University, Nanjing, 211198, China

**Keywords:** lunasin, proprotein convertase subtilisin/kexin type 9, low-density lipoprotein cholesterol, low-density lipoprotein receptor, hepatocyte nuclear factor-1α

## Abstract

Proprotein convertase subtilisin/kexin type 9 (PCSK9) is a serine protease which regulates serum low-density lipoprotein cholesterol (LDL-C) levels by promoting the degradation of the hepatic low-density lipoprotein receptor (LDLR), and has become an attractive therapeutic target for cholesterol lowering intervention. Lunasin, a 43-amino acid polypeptide initially isolated from soybean, has been previously proven to possess cholesterol lowering activity. Here we identified the down-regulation of PCSK9 expression by lunasin as one new mechanism that increased cell-surface LDLR level and enhanced LDL uptake *in vitro* and *in vivo*. Treatment of HepG2 cells with lunasin inhibited the expression of PCSK9 at mRNA and protein levels in a dose-and-time dependent manner via down-regulating hepatocyte nuclear factor-1α (HNF-1α), thereby contributing to increasing LDLR level and functionally enhancing LDL uptake. ApoE^−/−^ mice receiving lunasin administration by intraperitoneal injection at doses of 0.125∼0.5 μmol/kg·day for 4 weeks had significantly lower PCSK9 and higher LDLR levels in hepatic tissue, as well as remarkably reduced total-cholesterol (T-CHO) and LDL-C in blood as compared to mice in vehicle control group. Furthermore, we identified that LDLR expression was up-regulated by lunasin via PI3K/Akt-mediated activation of SREBP-2 in HepG2 cells. Taken together, our findings suggest that lunasin inhibits PCSK9 expression by down-regulating HNF-1α and enhances LDLR expression via PI3K/Akt-mediated activation of SREBP-2 pathway, thereby functionally enhances LDL uptake in HepG2 cells and in ApoE^−/−^ mice.

## INTRODUCTION

Hyperlipidemia, particularly the abnormally elevated plasma level of low-density lipoprotein cholesterol (LDL-C) plays a key role in causing atherosclerotic cardiovascular disease (CVD) [1, 2]. Thus, reduction of LDL-C constitutes the major approach for the prevention and treatment of CVD, and which could be achieved by both decreasing LDL-C synthesis and enhancing LDL-C clearance.

Statins are the most popular therapeutic drugs to decrease plasma LDL-C synthesis by inhibiting 3-hydroxy-3-methylglutaryl coenzyme A (HMG-CoA) reductase. However, for the clearance of circulating LDL-C in bloodstream, the major route is through hepatic LDL receptor (LDLR) mediated endocytosis in the liver. In this case, LDL-C is initially bound to hepatic cell-surface LDLR and internalized into clathrin-coated pits, and then degraded by lysosome. Thereafter, LDLR is recycled back to hepatic cell surface where it binds more LDL-C for endocytosis and degradation. It has been revealed that defect in LDLR is the most common cause of familial hypercholesterolemia (FH) and premature coronary artery diseases [3], and LDLR-adapter protein 1 (LDLRAP1) and low-density lipoprotein receptor-related protein 6 (LRP6) are required as co-receptors for an efficient endocytosis of LDLR-LDL-C complex in the liver [4, 5].

Proprotein convertase subtilisin/kexin type 9 (PCSK9) is a member of the mammalian proprotein convertase family of subtilisin-like serine endoproteases and is located mostly in adult liver hepatocytes, much less in the small intestine and kidney, and transiently expressed in the developing central nervous system [6]. It has been found that PCSK9 plays a pivotal role in the process of LDL catabolism because it can functionally promote the degradation of LDLR and prevents it from recycling to the membrane [7]. Accordingly, as an important regulator of lipoprotein metabolism, PCSK9 has become an attractive therapeutic target for cholesterol lowering intervention [8, 9]. To date, multiple approaches for inhibiting PCSK9 have been developed, which include interfering the binding of PCSK9 to LDLR by monoclonal antibodies and adnectins, inhibiting the synthesis of PCSK9 by gene silencing agents such as antisense oligonucleotides, small interfering RNA (siRNA) and small peptides, decreasing PCSK9 autocatalytic processing by small-molecule inhibitors [8, 10], as well as down-regulating the expression of PCSK9 by natural compounds such as berberine, curcumin and alginate oligosaccharide [11–13].

Lunasin, a 43- amino acid peptide which was initially isolated from the soybean and later identified in other plants such as wheat, amaranth, rye and barley etc [14], has been shown to possess potent anticancer activities *in vitro* and *in vivo* [15], and antioxidant as well as anti-inflammatory properties [16, 17]. Beyond that, previous studies have preliminarily identified lunasin as the active component in soy protein responsible for reducing LDL cholesterol by directly inhibiting the expression of HMG-CoA reductase and increasing the expression of LDLR [18].

However, to the best of our knowledge, there are no reports on the effects of lunasin on the function of PCSK9 thus far. In this work, we explored the regulatory effects of lunasin on the expression of PCSK9 *in vitro* and *in vivo*. As a result, we found that lunasin significantly suppressed the expression of PCSK9 in HepG2 cells and in ApoE^−/−^ mice, thereby contributing to the increased levels of LDLR and enhanced functions of LDL uptake in hepatic cells. Furthermore, we identified that up-regulation of LDLR by lunasin treatment was dependent on PI3K/Akt-mediated activation of SREBP-2 pathway.

## RESULTS

### Lunasin reduces PCSK9 expression at mRNA and protein levels in dose-and-time dependent manner in HepG2 cells

**Figure 1:**
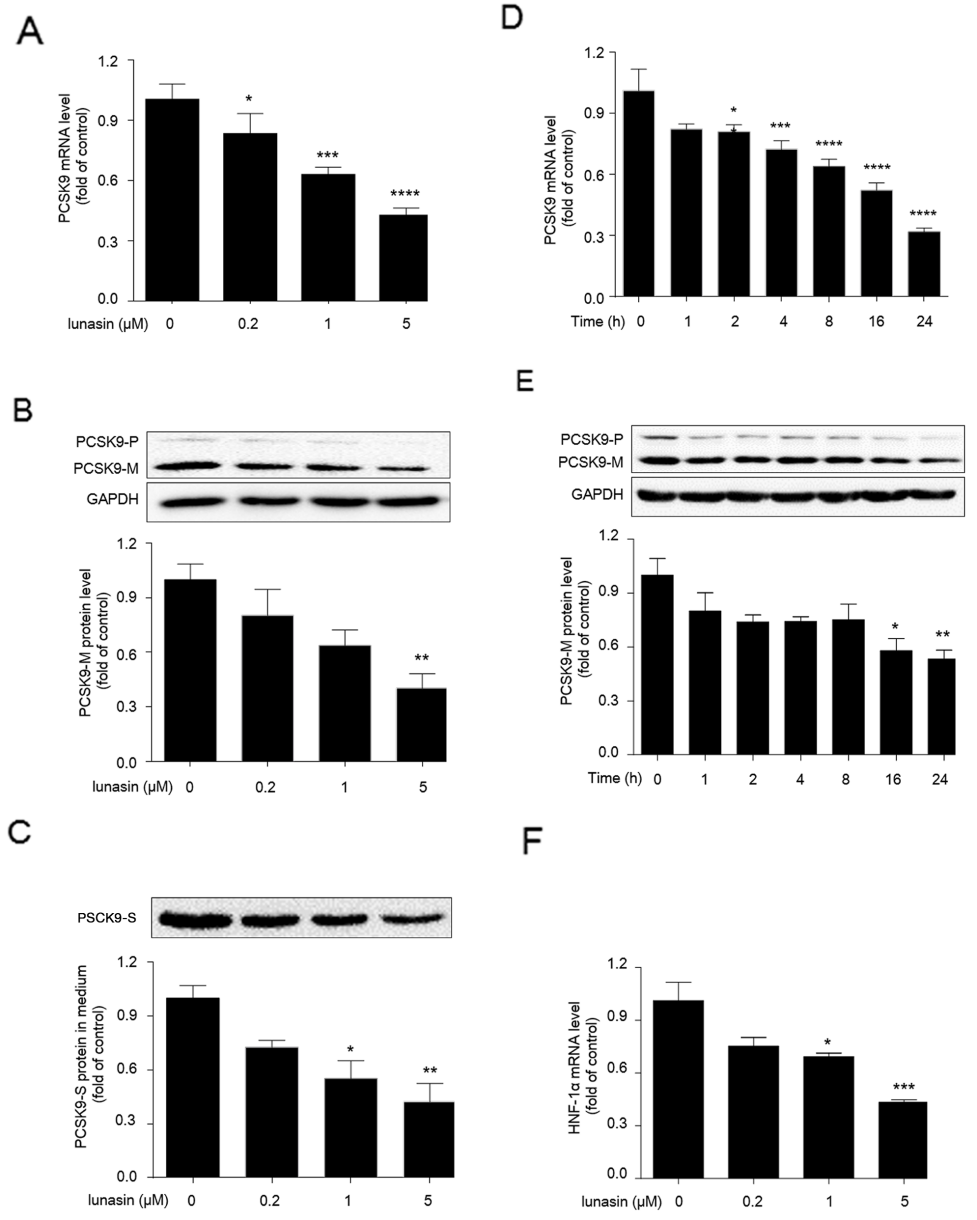
Lunasin inhibits the expression levels of PCSK9 and HNF-1α in HepG2 cells **(A, B, C)** HepG2 cells were treated with 0.2, 1, and 5 μM lunasin for 24 hrs, the mRNA and protein levels of intracellular precursor PCSK9 (PCSK9-P) and mature PCSK9 (PCSK9-M), as well as secreted PCSK9 (PCSK9-S) protein in culture medium were determined by qRT-PCR and Western blot, respectively. **(D, E)** HepG2 Cells were treated with 5 μM lunasin for various times (1, 2, 4, 8, 16, 24 hrs), and the levels of intracellular PCSK9 mRNA and protein were determined by qRT-PCR and Western blot. **(F)** HepG2 cells were treated with 0.2, 1, and 5μM lunasin for 24 hrs, and the mRNA levels of HNF-1α were analyzed by qRT-PCR. *p < 0.05, ^*^p < 0.01, ^***^p < 0.001, ^****^p < 0.0001 vs. control group. Results were given as the means ± SEM of three independent experiments.

HepG2 cells were cultured with increasing concentrations of lunasin (0, 0.2, 1 and 5 μM) for 24 hrs, then qRT-PCR and Western blot analyses were performed to measure PCSK9 expression at mRNA and protein levels, respectively. As shown in Figure 1, lunasin treatment inhibited PCSK9 expression at both mRNA (Figure 1A) and protein (Figure 1B) levels in HepG2 cells in a dose-dependent manner, and 5 μM of lunasin could remarkably suppress PCSK9 expression at mRNA (*p* < 0.0001) and protein (*p* < 0.01) levels in HepG2 cells as compared with the vehicle controls. To explore whether the inhibition of PCSK9 expression by lunasin led to the reduction of PCSK9 protein secreted from HepG2 cells, we measured the amounts of PCSK9 protein secreted in the cell culture media by Western blot analysis. As shown in Figure 1C, lunasin treatment also significantly reduced the amounts of PCSK9 protein secreted in the cell culture media in a dose-dependent manner. Besides, time-course experiment revealed that treatment of HepG2 cells with 5 μM lunasin for 0, 1, 2, 4, 8, 16, 24 hrs decreased PCSK9 expression at both mRNA (Figure 1D) and protein (Figure 1E) levels in a time-dependent manner. Thus, it was demonstrated that lunasin treatment significantly reduced both intracellular and secreted PCSK9 protein levels in HepG2 cells.

### Lunasin suppresses hepatocyte nuclear factor 1α (HNF-1α) transcription in HepG2 cells

HNF-1α has been previously identified as the predominant trans-activator for PCSK9 gene in HepG2 cells. To examine whether lunasin treatment affected the expression level of HNF-1α, HepG2 cells were treated with lunasin at concentrations of 0, 0.2, 1 and 5 μM for 24 hrs, and then qRT-PCR analysis was performed to detect the expression levels of HNF-1α. As a result, HNF-1α transcription was dramatically inhibited by 1 (*p* < 0.05) and 5 μM (*p* < 0.001) lunasin treatment in HepG2 cells (Figure 1F). Thus, the reduction of both intracellular and secreted PCSK9 proteins by lunasin treatment is likely to be associated with the suppression of HNF-1α in HepG2 cells.

### Lunasin increases LDLR level and enhances LDL uptake in HepG2 cells

**Figure 2:**
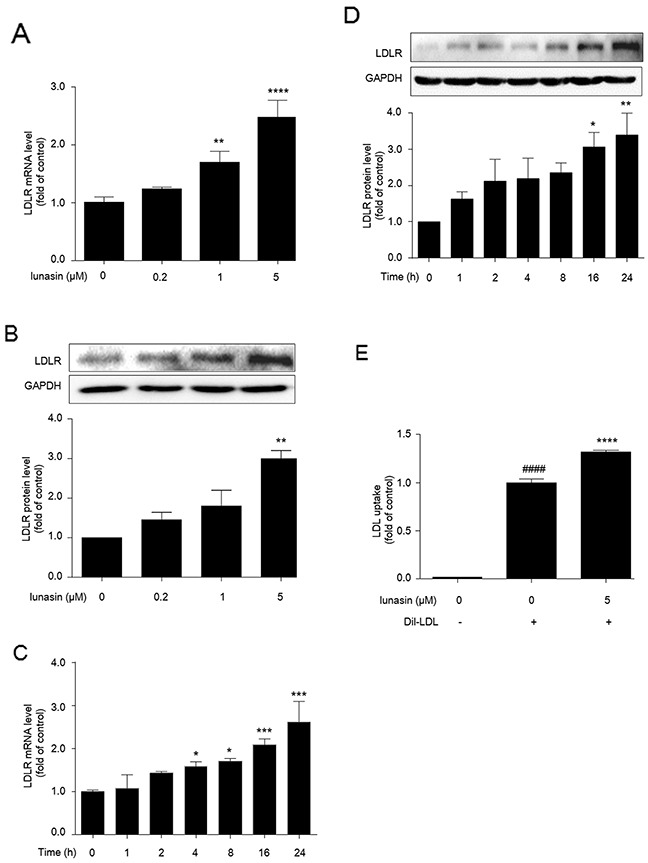
Lunasin up-regulates LDLR level and enhances LDL uptake in HepG2 cells **(A, B)** HepG2 cells were treated with 0.2, 1, and 5 μM lunasin for 24 hrs, the levels of LDLR mRNA and protein were determined by qRT-PCR and Western blot.**(C, D)** HepG2 Cells were treated with 5 μM lunasin for various times (1, 2, 4, 8, 16, 24 hrs), the levels of LDLR mRNA and protein were determined by qRT-PCR and Western blot. * *p* < 0.05, ^*^ *p* < 0.01, ^***^ *p* < 0.001, ^****^p < 0.0001 vs. vehicle control. **(E)** LDL uptake was assessed in HepG2 cells after treatment with 5μM lunasin for 24 hrs on a fluorescence plate reader. ^####^ *p* < 0.0001 vs. vehicle group; ^****^ *p* < 0.0001 vs. vehicle + 20 μg/mL Dil-LDL group. Results were given as the means ± SEM of three independent experiments.

**Figure 3:**
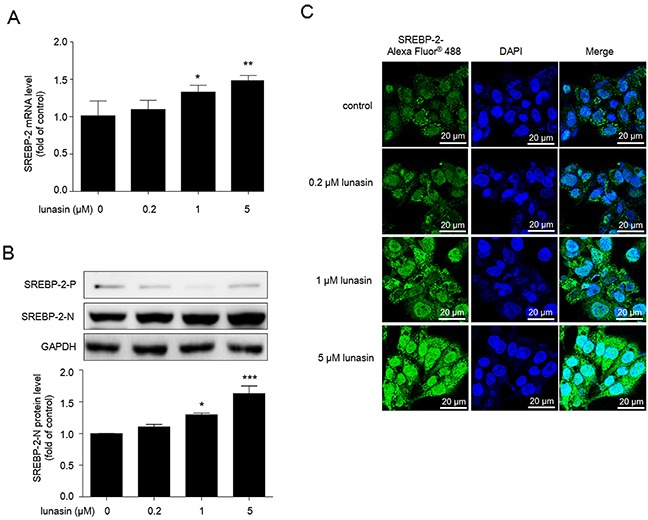
Lunasin increases nuclear form of SREBP-2 in HepG2 cells Cells were treated with 0.2, 1, and 5μM lunasin for 24 hrs. **(A)** The expression levels of SREBP-2mRNA were analyzed by qRT-PCR. **(B)** The expression of precursor SREBP-2 (SREBP-2-P) and nuclear SREBP-2 (SREBP-2-N) were analyzed by Western blot. **(C)** Immunofluorescence analysis used to detect the nuclear translocation of SREBP-2 in HepG2 cells. After treatment, cells were incubated with rabbit polyclonal anti-SREBP-2 antibody (1:200), and followed by incubation with Alexa Fluor® 488-conjugated goat anti-rabbit IgG (1:400) and DAPI (1 mM) to stain the nucleus. Then, cells were washed with PBS and images were taken by Zeiss LSM700 confocal microscopy at original magnification × 600. * *p* < 0.05, ^*^ *p* < 0.01 and ^***^ *p* < 0.001 vs. vehicle control group. Results are given as the means ± SEM of three independent experiments.

Following treatment of HepG2 cells with increasing concentrations of lunasin for 24 hrs, it was observed that the levels of LDLR mRNA and protein were increased in a dose-dependent manner (Figure 2A, 2B), and they were significantly increased 2.5-fold (*p* < 0.0001) and 3-fold (*p* < 0.01) by treatment with 5 μM of lunasin as compared to the vehicle control, respectively. On the other hand, after treatment with 5 μM lunasin for 0∼24 hrs, the amounts of LDLR mRNA and protein were increased in a time-dependent manner, and after treatment for 24 hrs they were increased 2-fold (*p* < 0.0001) (Figure 2C) and 3-fold (*p* < 0.01) (Figure 2D) as compared to the vehicle control, respectively. Further, functional analysis indicated that LDL uptake in HepG2 cells was vastly elevated after treatment with 5 μM of lunasin for 24 hrs as compared to the vehicle control (*p* < 0.0001) (Figure 2E).

### Lunasin up-regulates nuclear form of SREBP-2 in HepG2 cells

To examine the effects of lunasin treatment on the expression of SREBP-2, HepG2 cells were treated with 0∼5 μM lunasin for 24 hrs, the expression levels of SREBP-2 were measured by qRT-PCR analysis. The data indicated that lunasin significantly up-regulated the expression levels of SREBP-2 (Figure 3A) in a dose-dependent manner in HepG2 cells. Furthermore, the nuclear and precursor forms of SREBP-2 in HepG2 cells were examined by Western blot, and it was revealed that lunasin treatment significantly increased nuclear form of SREBP-2 in HepG2 cells (Figure 3B). Also, it was confirmed that lunasin treatment enhanced the translocation of SREBP-2 into nucleus in HepG2 cells by immunofluorescence analysis on the subcellular localization of SREBP-2 (Figure 3C).

### Lunasin enhances LDLR expression via PI3K/Akt-mediated SREBP-2 activation pathway in HepG2 cells

**Figure 4:**
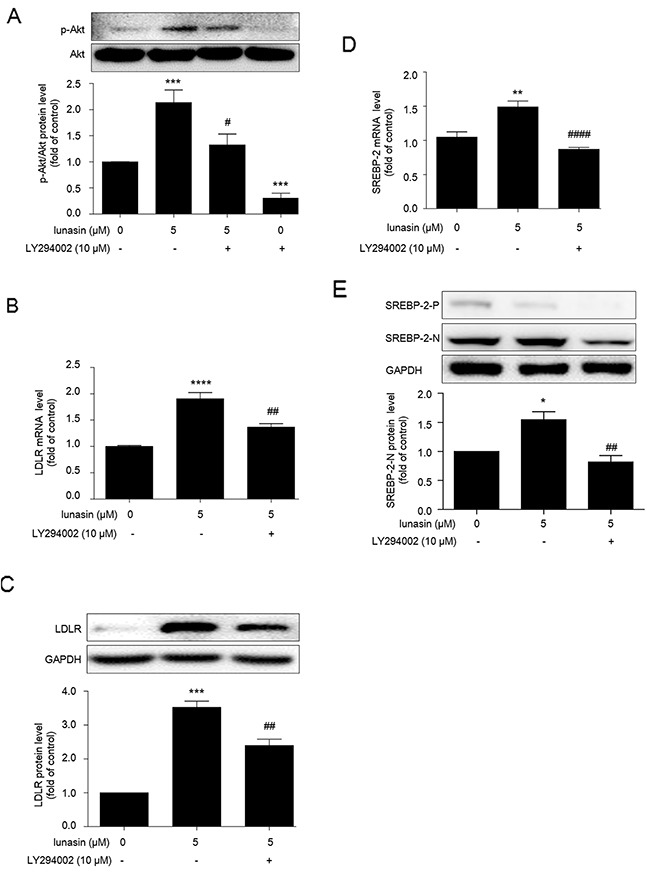
Lunasin enhances LDLR expression via PI3K/Akt-mediated SREBP-2 activation pathway in HepG2 cells HepG2 cells were pre-cultured with 10 μM LY294002 (an effective PI3K/Akt pathway inhibitor) for 2 hrs, then treated with or without 5 μM lunasin for 24 hrs. **(A)** Phosphorylation of Akt was analyzed by Western blot. **(B, C)** The levels of LDLR mRNA and protein were measured by qRT-PCR and Western blot, respectively. **(D, E)** The mRNA and protein levels of SREBP-2 were analyzed by qRT-PCR and Western blot, respectively. *p < 0.05,^*^ *p* < 0.01, ^***^ *p* < 0.001, ^****^ *p* < 0.0001 vs. vehicle control group; ^#^ *p* < 0.05, ^##^ *p* < 0.01, ^####^ *p* < 0.0001 vs. 5 μM lunasin group. Results are given as the means ± SEM of three independent experiments.

Phosphatidylinositol 3′ kinase (PI3K) and Akt are signaling kinases involved in cell survival and proliferation. Interestingly, recent advances suggested that PI3K/Akt also play crucial roles in the activation of SREBPs, master transcriptional regulators of lipid metabolism [19, 20]. Thus, we were prompted to evaluate the effects of lunasin treatment on this PI3K/Akt-mediated SREBP2 activation pathway. As shown in Figure 4A, lunasin treatment significantly increased Akt phosphorylation in HepG2 cells (*p* < 0.001), while it could be apparently restored by pretreatment of HepG2 cells with 10 μM PI3K inhibitor LY294002 prior to lunasin treatment (*p* < 0.05).

Besides, LY294002 counteracted the up-regulation of LDLR by lunasin at mRNA (*p* < 0.01) (Figure 4B) and protein (*p* < 0.01) (Figure 4C) levels, and significantly inhibited the increases in the amounts of SREBP-2 mRNA (*p* < 0.0001) (Figure 4D) and nuclear SREBP-2 protein (Figure 4E) induced by 5 μM lunasin treatment. These results suggest that PI3K/Akt-mediated SREBP-2 pathway is involved in the up-regulation of LDLR by lunasin treatment in HepG2 cells.

### Lunasin reduces plasma total cholesterol (T-CHO) and LDL cholesterol (LDL-C) in ApoE^−/−^mice

**Figure 5:**
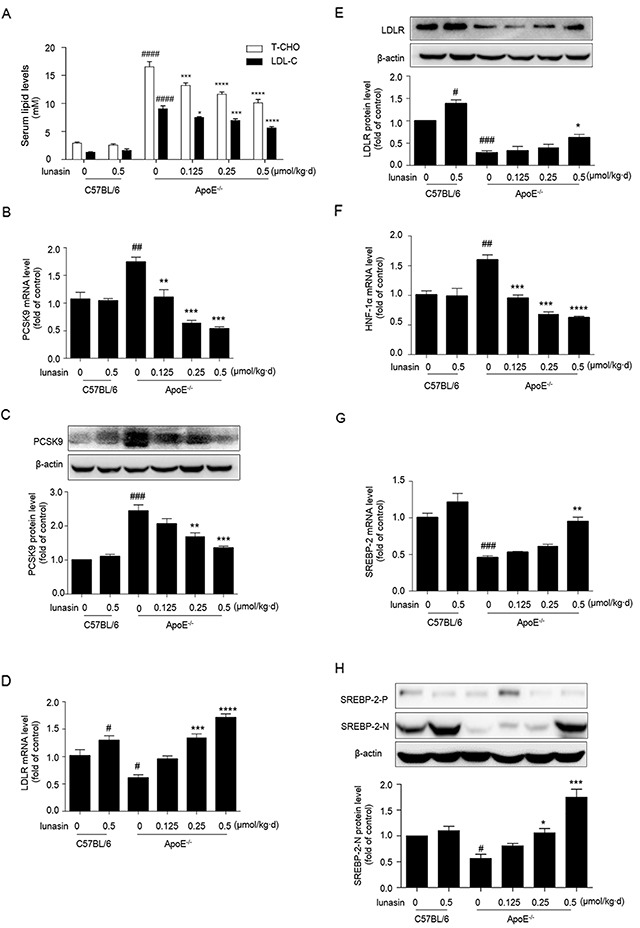
Lunasin reduces T-CHO, LDL-C in sera, decreases PCSK9 while increases the hepatic levels of LDLR, HNF-1α and nuclear form of SREBP-2 in ApoE^−/−^ mice ApoE^−/−^ mice fed with high fat diet were administered with lunasin by i. p. injection at dose of 0.125-0.5 μmol/kg·day for 4 weeks. **(A)** T-CHO, LDL-C serum lipid levels measured by biochemical kits. **(B, C, D, E)** The hepatic mRNA and protein levels of PCSK9 and LDLR analyzed by qRT-PCR and Western blot, respectively. **(F, G)** The hepatic mRNA levels of HNF-1α and SREBP-2 determined by qRT-PCR. **(H)** The hepatic precursor and nuclear forms of SREBP-2 analyzed by Western blot. ^#^ *p* < 0.05, ^##^ *p* < 0.01, ^###^ *p* < 0.001, ^####^ *p* < 0.0001 vs. C57BL/6 + vehicle (normal control); * *p* < 0.05, ^*^ *p* < 0.01, ^***^ *p* < 0.001, ^****^ *p* < 0.0001 vs. ApoE^−/−^ + vehicle (model control). Data were represented as the means ± SEM, n = 8 per group.

Given the effects of lunasin on PCSK9, LDLR and LDL uptake in HepG2 cells, we explored the *in vivo* anti-hyperlipidemia activity of lunasin in ApoE^−/−^ mice. It was observed that ApoE^−/−^ mice fed with high fat diet (HFD) exhibited greatly higher plasma T-CHO (*p* < 0.0001) and LDL-C (*p* < 0.0001) levels compared to C57BL/6 mice fed with normal diet. After administration with lunasin by i. p. injection at doses of 0.125-0.5 μmol/kg·day for 4 weeks, it was found that lunasin reduced the plasma T-CHO and LDL-C levels in a dose-dependent manner in ApoE^−/−^ mice fed with high fat diet (Figure 5A).

**Figure 6:**
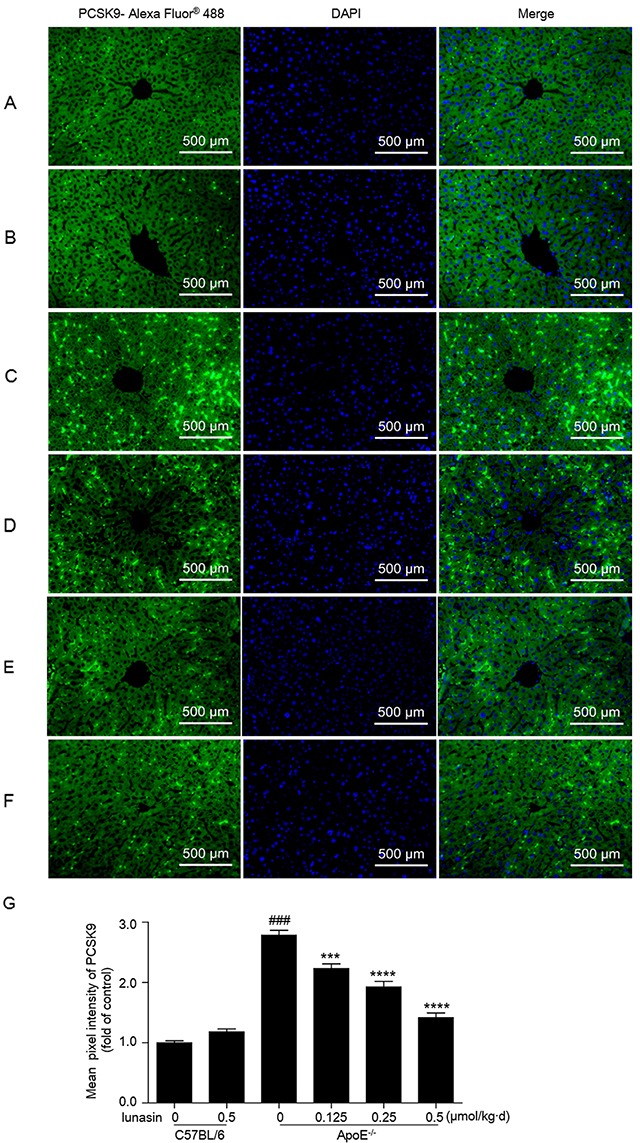
Lunasin decreases the level of PCSK9 secreted in hepatic tissues in ApoE^−/−^ mice by immunofluorescence analysis Hepatic tissue of mice was fixed in 4% paraformaldehyde and paraffin-embedded 4 μm sections subjected to immunofluorescence analysis. Representative images of liver paraffin sections stained for PCSK9 with Alexa Fluor^®^ 488 (green) and counterstained with DAPI to show cell nucleus (blue). **(A)** C57BL/6 + vehicle (normal control); **(B)** C57BL/6 + 0.5 μmol/kg·d lunasin; **(C)** ApoE^−/−^ + vehicle (model control); **(D)** ApoE^−/−^ + 0.125 μmol/kg·d lunasin; **(E)** ApoE^−/−^ + 0.25 μmol/kg·d lunasin; **(F)** ApoE^−/−^ + 0.5μmol/kg·d lunasin. **(G)** Quantitative analysis of PCSK9 image pixel intensity. ^####^ *p* < 0.0001 vs. C57BL/6 group; ^***^ *p* < 0.001 and ^****^ *p* < 0.0001 vs. ApoE^−/−^group. Data represented as the means ±SEM. The photomicrographs were taken by Zeiss AX10 fluorescence microcopy at original magnification × 200. Results are representative of three independent experiments with similar results.

Further, we evaluated the effects of lunasin treatment on the expression levels of PCSK9 and LDLR *in vivo*. As shown in Figure 5B, 5C, 5D, 5E, compared to normal control group (C57BL/6 mice fed common chow and i. p. administrated with vehicle), model control group (ApoE^−/−^ mice fed with HFD and i. p. administrated with vehicle) showed vastly high levels of PCSK9 mRNA and protein as well as lower levels of LDLR mRNA and protein in hepatic tissue, implying that the ApoE^−/−^ mouse model of dyslipidemia has been well-established. However, the levels of PCSK9 mRNA and protein were dramatically down-regulated, while the levels of LDLR mRNA and protein were significantly increased in a dose-dependent manner group in ApoE^−/−^ mice in response to lunasin treatment as compared to the model control. Besides, the expression of HNF-1α was strongly inhibited (Figure 5F) while the expression of SREBP-2 (Figure 5G) and the level of SREBP-2 nuclear protein (Figure 5H) were significantly increased in ApoE^−/−^ mice when treated with lunasin as compared to the model control. Additionally, it was observed that the levels of secreted PCSK9 in hepatic tissues of ApoE^−/−^ mice treated with lunasin were remarkably reduced in a dose-dependent manner as compared to the model control by immunofluorescence assay (Figure 6).

## DISCUSSION

**Figure 7:**
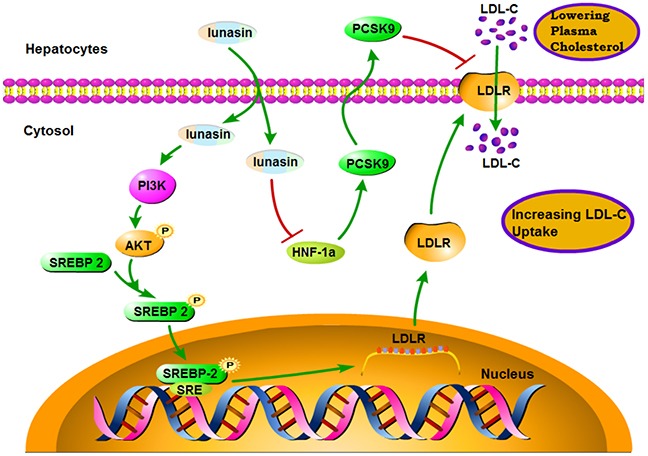
A schematic diagram of the molecular mechanism by which lunasin increases hepatic cell-surface LDLR level and functionally enhance LDL uptake in hepatocytes

In the present study, for the first time, we demonstrate that lunasin significantly suppresses the expression of PCSK9 via down-regulating HNF-1α in HepG2 cells, thereby contributing to the increased levels of LDLR and enhanced function of LDL uptake in hepatic cells, as well as lowered plasma LDL-cholesterol levels in ApoE^−/−^ mice fed with HFD. Furthermore, we identify that the up-regulation of LDLR by lunasin is dependent on PI3K/Akt-mediated activation of SREBP-2 pathway (Figure 7).

Previously, PCSK9 gene has been revealed to be located on chromosome 1p32 by chromosomal mapping [21]. The structure of the 692-aa human PCSK9 protein contains a prodomain (aa 31∼152) that is noncovalently associated with the mature protease chain (aa 153–692), keeping it in an enzymatically inactive state, and the catalytic subunit (aa 153–404) which is followed by a short hinge domain (Hi; aa 405–452) and the C-terminal CHRD (aa 453–692) composed of three repeats called M1, M2, and M3 [22]. The binding of PCSK9 to the first epidermal growth factor-like repeat (EGF-A) in the EGF homology domain of LDLR leads to internalization, impedes LDLR recycling and delivers LDLR to the lysosome for degradation [23], thus it makes PCSK9 a promising therapeutic target [24, 25].

A striking finding of this study is that lunasin as a novel PCSK9 inhibitor could significantly inhibit the gene expression of PCSK9 via down-regulating hepatocyte nuclear factor-1α (HNF-1α). We not only provided experimental evidences to indicate that lunasin dramatically down-regulated the levels of PCSK9 mRNA and mature protein both in time-dependent and dose-dependent manners in HepG2 cells, but also demonstrated that lunasin effectively decreased the levels of PCSK9 in ApoE^−/−^ mice. HNF-1α has been revealed to enhance the transcription of PCSK9 gene through a highly conserved HNF-1α binding site located 28bp upstream of the SRE-1 site in human PCSK9 promoter in HepG2 cells, which is associated with an increase in plasma PCSK9 levels in *vivo* [26, 27]. Thus, it can be hypothesized that drugs that down-regulate HNF-1α gene expression may play an important role in the reduction of PCSK9. To characterize the mechanism of the inhibitory effect of lunasin on PCSK9, we investigated whether HNF-1α could be down-regulated by lunasin treatment in HepG2 cells. As a result, lunasin dramatically suppressed the expression of HNF-1α in HepG2 cells. Notably, although the promoter region of the PCSK9 gene contains a sterol-regulatory element (SRE) site which can be transcriptionally activated by SREBP-2 [28], HNF-1α plays a critical role in PCSK9 gene transcription and regulation as HNF1 site mutation reduced PCSK9 promoter activity > 90% [27]. Thus, the suppression of HNF-1α by lunasin predominantly inhibited the expression of PCSK9 in spite of the up-regulation of SREBP-2.

On the other hand, we proved that lunasin remarkably up-regulated the levels of LDLR mRNA and protein in HepG2 cells, and which was associated with PI3K/Akt-mediated activation of SREBP-2 pathway. Sterol regulatory element binding proteins (SREBPs) are membrane bound transcription factors attached to the endoplasmic reticulum (ER), and which have been identified to function as master regulators of cholesterol and fatty acid synthesis [29, 30]. In mammalian, there are three SREBP isoforms (SREBP-1a, SREBP-1c and SREBP-2) which are encoded by two genes (SREBF1 and SREBF2), respectively [31]. Both SREBP-1 and SREBP-2 play important roles in the regulation of LDLR [32, 33]. In addition, *in vivo* studies suggested that both SREBP-1 isoforms show a relative preference for activating fatty acid synthesis but not cholesterol synthesis, while SREBP-2 is more specific to up-regulating the cholesterogenic genes [31, 34–38]. Notably, SREBPs cooperate with other DNA-binding transcription factors and coactivators, the maximal transcriptional activation requires additional DNA-binding proteins: nuclear factor-Y (NF-Y) and cAMP-response element binding protein (CREB) for the HMG-CoA reductase gene, and Sp1 for the LDL receptor gene [39, 40].

It has been known that SREBP regulation is associated with the phosphatidylinositol 3-kinase (PI3K)/Akt pathway [41], and Akt acutely activates the cholesterogenic transcription factor SREBP-2 via increasing SREBPs phosphorylation and subsequent ubiquitination [19, 42, 43]. Our data showed that the increase in the expression of SREBP-2 by lunasin treatment could be inhibited by LY294002, a PI3K/Akt pathway chemical inhibitor, thereby reducing the up-regulation of LDLR by lunasin treatment. These results suggested that PI3K/Akt-mediated SREBP-2 activation played a crucial role in the up-regulation of LDLR and enhancement of LDL uptake by lunasin treatment in hepatocytes, thereby decreasing the mice serum T-CHO and LDL-C *in vivo*.

Over the past decades, the use of statins to reduce LDL-C has successfully decreased the risk of cardiovascular events [44, 45], successfully in treating dyslipidemia as a major means for diminishing cardiovascular morbidity and mortality. However, statins have also been shown to increase the expression of PCSK9, which could contribute to the function limitation of statins in all situations [46, 47]. Therefore, as a PCSK9 natural inhibitor, lunasin might be used concomitantly with statins as a novel agent for LDL-C reduction.

In summary, our experimental data indicate that lunasin works in two ways to increase hepatic cell-surface LDLR level and thereby functionally enhance LDL uptake by both inhibiting PCSK9 expression via down-regulating HNF-1α and up-regulating LDLR expression via PI3K/Akt-mediated activation of SREBP-2 pathway in HepG2 cells and in ApoE^−/−^ mice. These findings suggest that lunasin could be a potential candidate for cholesterol homeostasis maintenance.

## MATERIALS AND METHODS

### Reagents and media

Lunasin was prepared by using recombinant DNA technology as previously described [48], and dissolved in saline. Minimum essential medium (MEM) and Opti-MEM as well as FBS were purchased from Gibco (Grand Island, NY, USA). Lipofectamine 3000 reagent was obtained from Invitrogen (California, Carlsbad, USA). Antibodies against PCSK9 and LDLR were obtained from abcam (Cambridge, UK). Anti-GAPDH and Anti-β-actin were obtained from Cell Signaling Technology (Danvers, MA, USA). LY294002, an effective PI3K/Akt pathway chemical inhibitor, was purchased from TargetMol (Boston, MA, USA). LDL labeled with 1, 1′-dioctadecyl – 3, 3, 3′, 3′-tetramethyl- indocarbocyanine perchlorate (Dil-LDL), which is highly fluorescent lipophilic dyes that diffuse into the hydrophobic portion of the LDL complex without affecting the LDL-specific binding of the apoprotein. Dil-LDL was obtained from Yiyuan Biotechnologies (Guangzhou, China). Dimethyl Sulphoxide (DMSO) and other chemicals were purchased from Amresco (Solon, OH, USA). Total cholesterol (CHO) and low-density lipoprotein cholesterol (LDL-C) assay kits were obtained from Nanjing Jiancheng Bioengineering Institute (Nanjing, China).

### Ethics statement

The animal experiment was carried out in accordance with the ethical standards and according to the Regulations of Experimental Animal Administrations issued by the State Committee of Science and Technology of the People's Republic of China.

### Cell culture

Human hepatic HepG2 cells were obtained from China Infrastructure of Cell Line Resources (Beijing, China) and cultured in complete medium consisting of MEM supplemented with penicillin (100 U/mL), streptomycin (100 μg/mL) and 10% Fetal Bovine Serum (FBS) in a humidified 5% CO_2_ atmosphere at 37°C. Opti-MEM media was used in the lunasin dose-response and time-course experiments to measure the amount of PCSK9 secreted into the culture media and LDLR expression.

### Lunasin dose-response and time-course experiments

Cells were seed in six well plates in media supplement with 10% FBS. When cells were grown to 70-80% confluence, changed the original medium into Opti-MEM media 1 hr before lunasin treatment. For dose-response experiments, vehicle or different concentrations of lunasin (0.2, 1, 5 μM) were added to the media and media/cells were harvested after 24 hrs. For time-course experiments, vehicle or 5 μM of lunasin were added to the media and cells/media were harvested after 1, 2, 4, 8, 16, 24 hrs. The results were measured by using Quantitative Reverse Transcriptase-PCR (qRT-PCR) and Western blot analysis.

### Measurement of Dil-LDL uptake

HepG2 cells were maintained in MEM supplemented with 10% FBS. The cells were seeded in 96 well black plates at a density of 1×10^4^ cells per well and grown to 70-80% confluence. Afterwards, cells were changed to serum-free Opti-MEM for 1hr and followed by incubation with 5 μM lunasin for 20 hrs. Then, 20 μg/mL Dil-LDL was added and incubated at 37°C in the dark for additional 4 hrs. Cells were extensively washed three times with PBS, and LDL uptake was determined on a fluorescence plate reader (Varioskan flash, Thermo) at an excitation wavelength of 520 nm and emission wavelength of 580 nm.

### Animals and drug administration

Six-week-old male C57BL/6 and ApoE^−/−^ mice obtained from Beijing Vital River Laboratory Animal Technology Co., Ltd. (Beijing, China) were housed in an air-conditioned animal house under a 12-hour light/dark cycle, 22-24°C room temperature, 40 ∼70% humidity, and had free access to rodent chow and tap water.

After an acclimatization period of 7 days, C57BL/6 mice divided randomly into two groups (n=8) were fed common chow and i. p. administrated with 0, 0.5 μmol/kg·day of lunasin in an application volume of 0.1 mL/10 g body weight, while ApoE^−/−^ mice divided randomly into four groups (n=8) were fed with high fat rodent diet containing 1.25% cholesterol (Research Diets, New Brunswick, NJ; Diet # D12108C) and i. p. administrated with 0, 0.125, 0.25, 0.5 μmol/kg·day of lunasin for 4 weeks, respectively. Each animal was used only in one experiment in order to exclude the influence of other tests. At the end of drug administration, after an overnight fast, blood samples were collected from mice eyes and rapidly moved into cold condition (0 ∼ 4°C) for 4 hrs. Each of liver tissues was separated into two parts, one was frozen immediately in liquid nitrogen for RNA and protein isolation, and the other was fixed overnight by 4% paraformaldehyde at 4°C for paraffin section. After ApoE^−/−^ mice were administrated with lunasin by i. p. injection at dose of 0.125∼0.5 μmol/kg·day for 4 weeks, the mRNA and protein levels of PCSK9 and LDLR in hepatic tissue were detected by qRT-PCR and Western blot, and the levels of secreted PCSK9 protein in ApoE^−/−^ mice hepatic tissue were detected by immunofluorescence analysis.

### Determination of serum lipid

Mice were fasted for 12 hrs prior to blood collection, blood was harvested into a centrifuge tube and allowed to obtain the serum that was separated by centrifugation at 3000 rpm, 4°C for 10 min. The serum total cholesterol (T-CHO) and LDL-C were measured by using the commercial kits in 96-well enzyme immunoassay plates.

### Immunofluorescence analysis

Nuclear translocation of the transcription factor SREBP-2 in HepG2 cells was detected by immunofluorescence using confocal microscopy. Briefly, cells were fixed in 4% para-formaldehyde/PBS for 30 min and followed by incubation with permeabilization solution (0.1% Triton-100/PBS) for 30 min at room temperature. Cells were first blocked using 10% rabbit serum/PBST for 1 hr and incubated with goat polyclonal anti-SREBP-2 antibody (1:200) overnight at 4°C, followed by incubation with Alexa Fluor^®^ 488-conjugated goat anti-rabbit IgG (1:400) for 1 hr at room temperature and counterstained with DAPI (KeyGEN BioTECH, Nanjing, China) to show cell nucleus. After incubation, cells were washed with PBS and images were taken by Zeiss LSM700 confocal microscopy (Zeiss, Oberkochen, Germany). Negative control was performed by omitting the primary antibody and revealed no labeling (data not shown).

For histological analysis, liver tissues of all groups were fixed in 4% para-formaldehyde/PBS at 4°C overnight, embedded in paraffin and sliced at 4 μm thickness. After deparaffinization and hydration, tissue sections were pretreated by heating for 20 min in sodium citrate solution (0.01 M, pH 6.0) in a 95°C water bath for the antigen retrieval. Prior to blocking with 10% goat serum/TBST for 15 min, tissue sections were treated with 3% H_2_O_2_ for 20 min, then sequentially incubated with rabbit anti-PCSK9 polyclonal antibody (1:100) at 4°C overnight, followed by incubating with Alexa Fluor^®^ 488-conjugated goat anti-rabbit IgG (1:400) for 1 hr at 37°C in the dark. After rinsing with PBS, sections were counterstained with DAPI (KeyGEN BioTECH, Nanjing, China) to show cell nucleus, rinsed three times in PBS, mounted with glycerol/PBS (1:1) and photographed by Zeiss AX10 fluorescence microcopy (Zeiss, Oberkochen, Germany). Negative control was performed by omitting the primary antibody and revealed no labeling (data not shown).

### Western blot analyses of LDLR, PCSK9 in HepG2 cells and in mice hepatic tissues

Western blot analysis was performed to measure the protein expressions in medium and cells as well as tissues as previously described [49, 50]. HepG2 cells were washed three times in ice-cold PBS and then lysed in cold RIPA lysis buffer compounding 1 mM PMSF (Amresco, US). For secreted protein preparations, the Opti-MEM medium of the cultured HepG2 cells was collected, centrifuged to remove the cells, and concentrated by ultrafiltration tube (Merck Millipore, Darmstadt, Germany) as previously described [51]. For the liver tissues, samples were added with cold lysis buffer according to the weight of liver tissues and homogenized by using glass homogenizer. The lysates or homogenates were centrifuged and the supernatant was harvested. Total protein concentrations were determined with BCA protein assay kit (Biouniquer, Beijing, China). An equal amount of proteins from each sample were loaded on 10% sodium dodecyl sulfate-polyacrycamide gel (SDS-PAGE) and separated. The separated proteins were electrophoretically transferred onto a 0.22 μm PVDF membrane (Merck Millipore, Darmstadt, Germany) followed by blocking with a solution of 0.1% Tween 20/TBS containing 5 % nonfat milk for 1hr at room temperature. Subsequently, the membranes were incubated with corresponding primary anti-LDLR, anti-PCSK9, anti-SREBP-2, anti-GAPDH antibodies at 4°C overnight, incubated with peroxidase-conjugated secondary antibodies (1:5000) for 1 hr at room temperature. Finally, membranes exposed with ECL (Thermo Scientific, Massachusetts, USA) after another 3 times of washing with TBST for 5 min. Quantification of protein bands was obtained using Image J software.

### Quantitative reverse transcriptase-PCR (qRT-PCR) analysis of LDLR, PCSK9, HNF-1α and SREBP-2 in HepG2 cells and in ApoE^−/−^ mice

HepG2 cells were lysed with RNAiso plus reagent (Takara, Japan). Total RNA was isolated in accordance with the manufacturer's protocols and resuspended in RNAase free water. Then, isolated RNA was quantified by measuring absorption at 260 nm with NanoDrop 2000/2000 c (Thermo Scientific). RNA integrity was confirmed by ethidium bromide staining of agarose gel electrophoresis. The cDNA was then synthesized from 1 μg total RNA using a reverse transcription kit (Takara, Japan) according to the recommendations of the manufacturer. qRT-PCR was performed with 50∼100 ng cDNA template and specific primers in the MX3000PTM qRT-PCR instrument (Agilent scientific) using SYBR® Premix Ex Taq ™II (Takara, Japan) according to the manufacturer's protocols. Primers for each gene were showed in Table 1. Target mRNA expression levels in each sample were normalized to the housekeeping gene GAPDH. The 2^−ΔΔCt^ method was used to calculate relative mRNA expression levels [52]. Each run was completed with a melting curve analysis to confirm the specificity of amplification and lack of primer dimers.

**Table 1:** Primer sequences used for real-time PCR

Gene	Forward primer (5′ → 3′)	Reverse primer(5′ → 3′)	Amplicon size (bp)
PCSK9	AGGGGAGGACATCATTGGTG	CAGGTTGGGGGTCAGTACC	229
HNF-1α	AGGACGAGACGGACGACGAT	AGTGCCCTTGTTGAGGTGTT	245
LDLR	GAACCCATCAAAGAGTGCG	TCTTCCTGACCTCGTGCC	313
SREBP-2	GTCCTGAGCGTCTTTGTGA	CCAGGCAGGTTTGTAGGTT	160
β-actin	CTCTTCCAGCCTTCCTTCCT	CAGGGCAGTGATCTCCTTCT	180
Mus-β-actin	GTGACGTTGACATCCGTAAAGA	GCCGGACTCATCGTACTCC	245
Mus-PCSK9	CAGAGGTCATCACAGTCGGG	GGGGCAAAGAGATCCACACA	100
Mus-HNF-1α	GAGCCTGAATCGAGCAGAAC	AGCCTTCTCTGGACACCTGA	184
Mus-LDLR	GTATGAGGTTCCTGTCCATC	CCTCTGTGGTCTTCTGGTAG	144
Mus-SREBP-2	CCAAAGAAGGAGAGAGGCGG	CGCCAGACTTGTGCATCTTG	129

### Statistical analysis

Duplicates were used in all experiments and all experiments were repeated at least three times, and values are reported as the means ± SEM, n refers to the numbers of experimental animals. Statistical analysis was performed using GraphPad Prism software (version 6.0). Significant differences between control and treatment groups were assessed by one-way ANOVA with proper posttest or two-way ANOVA for multiple comparisons. Statistical significance is defined by *p* < 0.05.

## Abbreviations

PCSK9: proprotein convertase subtilisin/kexin type 9
PCSK9-M: mature PCSK9
PCSK9-P: precursor PCSK9
PCSK9-S: secreted PCSK9
LDL: low density lipoprotein
LDL-C: low density lipoprotein cholesterol
LDLR: low density lipoprotein receptor
VLDL: very low density lipoprotein
CVD: cardiovascular disease
CM: chylomicron
HMG-CoA reductase: 3-hydroxy-3-methylglutaryl coenzyme A reductase
LDLRAP1: LDLR-adapter protein 1
LRP6: low-density lipoprotein receptor-related protein 6
PBS: phosphate-buffered saline
MEM: minimum essential medium
FBS: fetal bovine serum
T-CHO: total-cholesterol
HNF-1α: hepatocyte nuclear factor-1α
PI3K: phosphatidylinositol 3 kinase
FBS: fetal bovine serum
NS: normal saline
DMSO: dimethyl sulphoxide
HFD: high fat diet
i. p.: intraperitoneally
SREBP: sterol regulatory element binding protein
SREBP-2-P: precursor SREBP-2
SREBP-2-N: nuclear SREBP-2

## References

[1] Tietge UJ (2014). Hyperlipidemia and cardiovascular disease: inflammation, dyslipidemia, and atherosclerosis. Curr Opin Lipidol.

[2] Rader DJ, Daugherty A (2008). Translating molecular discoveries into new therapies for atherosclerosis. Nature.

[3] Go GW, Mani A (2012). Low-density lipoprotein receptor (LDLR) family orchestrates cholesterol homeostasis. Yale Journal of Biology & Medicine.

[4] Go GW (2015). Low-density lipoprotein receptor-related protein 6 (LRP6) is a novel nutritional therapeutic target for hyperlipidemia, non-alcoholic fatty liver disease, and atherosclerosis. Nutrients.

[5] Alvarez ML, Khosroheidari M, Eddy E, Done SC (2015). MicroRNA-27a decreases the level and efficiency of the LDL receptor and contributes to the dysregulation of cholesterol homeostasis. Atherosclerosis.

[6] Seidah NG, Benjannet S, Wickham L, Marcinkiewicz J, Jasmin SB, Stifani S, Basak A, Prat A, Chretien M The secretory proprotein convertase neural apoptosis-regulated convertase 1 (NARC-1): liver regeneration and neuronal differentiation. Proceedings of the National Academy of Sciences.

[7] Abifadel M, Varret M, Rabès JP, Allard D, Ouguerram K, Devillers M, Cruaud C, Benjannet S, Wickham L, Erlich D (2003). Mutations in PCSK9 cause autosomal dominant hypercholesterolemia. Nature Genetics.

[8] Norata GD, Tibolla G, Catapano AL (2014). Targeting PCSK9 for hypercholesterolemia. Annu Rev Pharmacol Toxicol.

[9] Della Badia LA, Elshourbagy NA, Mousa SA (2016). Targeting PCSK9 as a promising new mechanism for lowering low-density lipoprotein cholesterol. Pharmacol Ther.

[10] Milionis H, Liamis G, Elisaf M (2015). Proprotein convertase subtilisin kexin 9 inhibitors: next generation in lipid-lowering therapy. Expert Opin Biol Ther.

[11] Cameron J, Ranheim T, Kulseth MA, Leren TP, Berge KE (2008). Berberine decreases PCSK9 expression in HepG2 cells. Atherosclerosis.

[12] Tai MH, Chen PK, Chen PY, Wu MJ, Ho CT, Yen JH (2014). Curcumin enhances cell-surface LDLR level and promotes LDL uptake through downregulation of PCSK9 gene expression in HepG2 cells. Mol Nutr Food Res.

[13] Yang JH, Bang MA, Jang CH, Jo GH, Jung SK, Ki SH (2015). Alginate oligosaccharide enhances LDL uptake via regulation of LDLR, PCSK9 expression. J Nutr Biochem.

[14] Hernández-Ledesma B, Hsieh CC, Lumen BOD (2009). Lunasin, a novel seed peptide for cancer prevention. Peptides.

[15] Hernández-Ledesma B, Hsieh CC, de Lumen BO (2013). Chemopreventive properties of Peptide Lunasin: a review. Protein & Peptide Letters.

[16] Cam A, Sivaguru M, Gonzalez dME (2013). Endocytic mechanism of internalization of dietary peptide lunasin into macrophages in inflammatory condition associated with cardiovascular disease. PLos One.

[17] Garcíanebot MJ, Recio I, Hernándezledesma B (2014). Antioxidant activity and protective effects of peptide lunasin against oxidative stress in intestinal Caco-2 cells. Food & Chemical Toxicology.

[18] Galvez AF (2012). Identification of lunasin as the active component in soy protein responsible for reducing LDL cholesterol and risk of cardiovascular disease. Circulation.

[19] Du X, Kristiana I, Wong J, Brown AJ (2006). Involvement of Akt in ER-to-Golgi transport of SCAP/SREBP: a link between a key cell proliferative pathway and membrane synthesis. Molecular Biology of the Cell.

[20] Krycer JR, Sharpe LJ, Luu W, Brown AJ (2010). The Akt–SREBP nexus: cell signaling meets lipid metabolism. Trends in Endocrinology & Metabolism Tem.

[21] Seidah NG, Benjannet S, Wickham L, Marcinkiewicz J, Jasmin SB, Stifani S, Basak A, Prat A, Chretien M (2003). The secretory proprotein convertase neural apoptosis-regulated convertase 1 (NARC-1): liver regeneration and neuronal differentiation. Proc Natl Acad Sci U S A.

[22] Seidah NG, Abifadel M, Prost S, Boileau C, Prat A (2017). The Proprotein Convertases in Hypercholesterolemia and Cardiovascular Diseases: Emphasis on Proprotein Convertase Subtilisin/Kexin 9. Pharmacol Rev.

[23] Zhang DW, Lagace TA, Garuti R, Zhao Z, Mcdonald M, Horton JD, Cohen JC, Hobbs HH (2007). Binding of proprotein convertase subtilisin/kexin type 9 to epidermal growth factor-like repeat A of low density lipoprotein receptor decreases receptor recycling and increases degradation. Journal of Biological Chemistry.

[24] Seidah NG, Prat A (2012). The biology and therapeutic targeting of the proprotein convertases. Nature Reviews Drug Discovery.

[25] Seidah NG (2009). PCSK9 as a therapeutic target of dyslipidemia. Expert Opinion on Therapeutic Targets.

[26] Shende VR, Wu M, Singh AB, Dong B, Kan CF, Liu J (2015). Reduction of circulating PCSK9 and LDL-C levels by liver-specific knockdown of HNF1α in normolipidemic mice. Journal of Lipid Research.

[27] Li H, Dong B, Park SW, Lee HS, Chen W, Liu J (2009). Hepatocyte nuclear factor 1alpha plays a critical role in PCSK9 gene transcription and regulation by the natural hypocholesterolemic compound berberine. J Biol Chem.

[28] Jeong HJ, Lee HS, Kim KS, Kim YK, Yoon D, Park SW (2008). Sterol-dependent regulation of proprotein convertase subtilisin/kexin type 9 expression by sterol-regulatory element binding protein-2. J Lipid Res.

[29] Shimano H (2001). Sterol regulatory element-binding proteins (SREBPs): transcriptional regulators of lipid synthetic genes. Progress in Lipid Research.

[30] Shimano H (2002). Sterol regulatory element-binding protein family as global regulators of lipid synthetic genes in energy metabolism. Vitamins and Hormones.

[31] Horton JD, Shimomura I, Brown MS, Hammer RE, Goldstein JL, Shimano H (1998). Activation of cholesterol synthesis in preference to fatty acid synthesis in liver and adipose tissue of transgenic mice overproducing sterol regulatory element-binding protein-2. Journal of Clinical Investigation.

[32] Hua X, Yokoyama C, Wu J, Briggs MR, Brown MS, Goldstein JL, Wang X (1993). SREBP-2, a second basic-helix-loop-helix-leucine zipper protein that stimulates transcription by binding to a sterol regulatory element. Proceedings of the National Academy of Sciences of the United States of America.

[33] Yokoyama C, Wang X, Briggs MR, Admon A, Wu J, Hua X, Goldstein JL, Brown MS (1993). SREBP-1, a basic-helix-loop-helix-leucine zipper protein that controls transcription of the low density lipoprotein receptor gene. Cell.

[34] Shimano H, Horton JD, Hammer RE, Shimomura I, Brown MS, Goldstein JL (1996). Overproduction of cholesterol and fatty acids causes massive liver enlargement in transgenic mice expressing truncated SREBP-1a. Journal of Clinical Investigation.

[35] Shimano H, Horton JD, Shimomura I, Hammer RE, Brown MS, Goldstein JL (1997). Isoform 1c of sterol regulatory element binding protein is less active than isoform 1a in livers of transgenic mice and in cultured cells. Journal of Clinical Investigation.

[36] Shimano H, Yahagi N, Amemiyakudo M, Hasty AH, Osuga J, Tamura Y, Shionoiri F, Iizuka Y, Ohashi K, Harada K (1999). Sterol regulatory element-binding protein-1 as a key transcription factor for nutritional induction of lipogenic enzyme genes. Journal of Biological Chemistry.

[37] Pai J, Guryev O, Brown MS, Goldstein JL (1998). Differential Stimulation of Cholesterol and Unsaturated Fatty Acid Biosynthesis in Cells Expressing Individual Nuclear Sterol Regulatory Element-binding Proteins.

[38] Horton JD, Goldstein JL, Brown MS (2002). SREBPs: activators of the complete program of cholesterol and fatty acid synthesis in the liver. Journal of Clinical Investigation.

[39] Edwards PA, Tabor D, Kast HR, Venkateswaran A (2000). Regulation of gene expression by SREBP, SCAP. Biochim Biophys Acta.

[40] Espenshade PJ (2006). SREBPs: sterol-regulated transcription factors. J Cell Sci.

[41] Manning BD, Cantley LC (2007). AKT/PKB Signaling: Navigating Downstream. Cell.

[42] Luu W, Sharpe LJ, Stevenson J, Brown AJ (2012). Akt acutely activates the cholesterogenic transcription factor SREBP-2. Biochimica Et Biophysica Acta.

[43] Sundqvist A, Bengoecheaalonso MT, Ye X, Lukiyanchuk V, Jin J, Harper JW, Ericsson J (2005). Control of lipid metabolism by phosphorylation-dependent degradation of the SREBP family of transcription factors by SCF(Fbw7). Cell Metabolism.

[44] Baigent C, Keech A, Kearney PM, Blackwell L, Buck G, Pollicino C, Kirby A, Sourjina T, Peto R, Collins R (2005). Efficacy and safety of cholesterol-lowering treatment: prospective meta-analysis of data from 90,056 participants in 14 randomised trials of statins. Lancet.

[45] Genser B, März W (2006). Low density lipoprotein cholesterol, statins and cardiovascular events: a meta–analysis. Clinical Research in Cardiology.

[46] Careskey HE, Davis RA, Alborn WE, Troutt JS, Cao G, Konrad RJ (2008). Atorvastatin increases human serum levels of proprotein convertase subtilisin/kexin type 9. Journal of Lipid Research.

[47] Dubuc G, Chamberland A, Wassef H, Davignon J, Seidah NG, Bernier L, Prat A (2004). Statins upregulate PCSK9, the gene encoding the proprotein convertase neural apoptosis-regulated convertase-1 implicated in familial hypercholesterolemia. Arteriosclerosis Thrombosis & Vascular Biology.

[48] Tian Q, Zhang P, Gao Z, Li H, Bai Z, Tan S (2017). Hirudin as a Novel Fusion Tag for Efficient Production of Lunasin in Escherichia Coli. Prep Biochem Biotechnol.

[49] Dong B, Kan CF, Singh AB, Liu J (2013). High-fructose diet downregulates long-chain acyl-CoA synthetase 3 expression in liver of hamsters via impairing LXR/RXR signaling pathway. Journal of Lipid Research.

[50] Dong B, Singh AB, Fung C, Kan K, Liu J (2014). CETP inhibitors downregulate hepatic LDL receptor and PCSK9 expression in vitro and in vivo through a SREBP2 dependent mechanism. Atherosclerosis.

[51] Tai MH, Chen PK, Chen PY, Wu MJ, Ho CT, Yen JH (2014). Curcumin enhances cell-surface LDLR level and promotes LDL uptake through downregulation of PCSK9 gene expression in HepG2 cells. Molecular Nutrition & Food Research.

[52] Livak KJ, Schmittgen TD (2001). Analysis of relative gene expression data using real-time quantitative PCR and the 2(−Delta Delta C(T)) Method. Methods.

